# Dosimetric impact of contour editing on CT and MRI deep‐learning autosegmentation for brain OARs

**DOI:** 10.1002/acm2.14345

**Published:** 2024-04-25

**Authors:** Nouf M. Alzahrani, Ann M. Henry, Anna K. Clark, Bashar M. Al‐Qaisieh, Louise J. Murray, Michael G. Nix

**Affiliations:** ^1^ Department of Diagnostic Radiology King Abdulaziz University Jeddah Saudi Arabia; ^2^ School of Medicine University of Leeds Leeds UK; ^3^ Department of Medical Physics and Engineering St James's University Hospital Leeds UK; ^4^ Department of Clinical Oncology St James's University Hospital Leeds UK

**Keywords:** autosegmentation, brain cancer, CT scans, deep learning, dosimetric evaluation, MRI scans, organs at risk

## Abstract

**Purpose:**

To establish the clinical applicability of deep‐learning organ‐at‐risk autocontouring models (DL‐AC) for brain radiotherapy. The dosimetric impact of contour editing, prior to model training, on performance was evaluated for both CT and MRI‐based models. The correlation between geometric and dosimetric measures was also investigated to establish whether dosimetric assessment is required for clinical validation.

**Method:**

CT and MRI‐based deep learning autosegmentation models were trained using edited and unedited clinical contours. Autosegmentations were dosimetrically compared to gold standard contours for a test cohort. D1%, D5%, D50%, and maximum dose were used as clinically relevant dosimetric measures. The statistical significance of dosimetric differences between the gold standard and autocontours was established using paired Student's *t*‐tests. Clinically significant cases were identified via dosimetric headroom to the OAR tolerance. Pearson's Correlations were used to investigate the relationship between geometric measures and absolute percentage dose changes for each autosegmentation model.

**Results:**

Except for the right orbit, when delineated using MRI models, the dosimetric statistical analysis revealed no superior model in terms of the dosimetric accuracy between the CT DL‐AC models or between the MRI DL‐AC for any investigated brain OARs. The number of patients where the clinical significance threshold was exceeded was higher for the optic chiasm D1% than other OARs, for all autosegmentation models. A weak correlation was consistently observed between the outcomes of dosimetric and geometric evaluations.

**Conclusions:**

Editing contours before training the DL‐AC model had no significant impact on dosimetry. The geometric test metrics were inadequate to estimate the impact of contour inaccuracies on dose. Accordingly, dosimetric analysis is needed to evaluate the clinical applicability of DL‐AC models in the brain.

## 1 INTRODUCTION

With the advancement of technology and the increasing number of brain cancer patients,^1^ clinical use of brain OARs deep learning autocontouring (DL‐AC) models in the radiotherapy department has become attractive. It promises to improve the standardization and efficiency of organ‐at‐risk (OAR) contouring.^2^ However, appropriate evaluation of contour quality and clinical acceptability is a challenge. While geometric evaluation is straightforward, generalizable, and quantitative, its connection to clinical impact is difficult to establish.^3^, ^4^ The most popular methods for evaluating autosegmentation geometric quality are the Dice similarity coefficient (DSC) and distance‐to‐agreement metrics (DTA).^3^ Overlap metrics can be sensitive to structure size and are frequently poor predictors of impact on clinically relevant dosimetric parameters.^3^, ^4^ Conversely, dosimetric analysis depends on local treatment protocols and clinical criteria, as well as individual patient anatomy and dose distributions, making it harder to draw general conclusions about model performance.

Researchers have reported that the optimal evaluation method depends on the aim of autosegmentation.^2^ Where autocontours will be checked and edited by human operators, geometric or editing‐time based analysis may be sufficient, although dosimetric analysis can inform operators about the clinical significance of editing and hence maximize time savings.^2^ If contours will be used directly, for example, in online adaptive therapy, with minimal or zero human intervention, a higher bar of both geometric and dosimetric testing is needed to ensure patient safety.

Therefore, to determine the clinical feasibility of autosegmentation for radiation treatment planning and delivery, several evaluation strategies, including geometric, dosimetric, and physician assessment, are ideally required.^2^

Dosimetric evaluation is most directly linked to clinical relevance.^4^ However, this analysis requires treatment planning data.^4^ Also, there is no standard method or agreed threshold of acceptability for dosimetric variation.^5^ Accordingly, there is little research on the dosimetric effects of contour variations between manual and autosegmentation, and even less on the dosimetric consequences of editing contours either before model training (as here) or post autosegmentation.^6^

Recent research^2^, ^3^ raises questions about the correlation between common geometric measures, dose planning statistics, and clinical acceptability of OAR contours. Hence, it is difficult to establish whether a segmentation model is clinically usable in a specific clinical scenario, sufficiently limiting the risk of overexposing normal tissue and allowing the precise delivery of RT dose to targets.

This study investigates the dosimetric impact of autocontouring OARs in the brain, in the context of RT for common brain cancers. This work is built upon a geometric evaluation which was previously published and hence focusses on the clinically relevant dosimetric aspects.^7^ The correlation of dosimetry with the geometric accuracy of MRI and CT‐based DL‐AC models, established previously,^6^, ^8^, ^9^ is also addressed. Further, we determine the dosimetric impact of editing clinical contours to gold standard quality before training CT and MRI DL‐AC models. Previous geometric analysis showed that DL‐AC models trained with edited clinical contours successfully generated more segmentations than the models trained with unedited clinical contours. Also, editing contours on MRI before model training improved the geometric performance.^7^ However, generating gold standard contours is a time‐consuming process that may require several clinicians, it severely limits the amount of high‐quality labeled data available for model training. Also, there are no specific guidelines on the level of editing required, and the trade‐off between training data quantity and quality. While DL‐Autocontouring delineations are usually checked or edited before use, poorer quality results from model involving limited unedited data may cause loss of efficiency and increase risk. However, if found editing contours to be unnecessary before training the DL‐AC model, larger amounts of un‐curated data could be a more efficient route to high‐quality autosegmentation models for OARs in RT, particularly for MRI models, where limited data with equivalent sequences is available.

Understanding the impact of autosegmentation on RT dosimetry could also improve guidance for the critical assessment and editing of autocontours in clinical practice, maximizing time‐efficiency gains while avoiding an increased risk of toxicity from overexposing OARs.

Overdosing brain OARs can lead to, for example, visual and hearing deficits, making understanding of OAR segmentation accuracy a critical requirement in delivering high‐quality RT.^10^

Previous studies of autocontouring for brain OARs using deep learning relied only on geometric assessment.^7^, ^11^, ^12^, ^13^ By evaluating the correlation between geometric and dosimetric measures, we aim to establish whether geometric assessment alone is sufficient to evaluate brain OAR autosegmentation tools or whether an additional dosimetric evaluation is also needed.

Regarding other treatment sites (thoracic, esophageal, and head and neck), several studies have assessed the dosimetric impact of deep learning segmentation.^6^, ^8^, ^9^ Correlations between the geometric and dosimetric measures in thoracic and head and neck OARs have not been identified.^6^, ^8^ In contrast, a study investigating esophageal OARs revealed that DSC and OAR dose had a statistically significant overall correlation, although this correlation was not always present at the level of individual patients or OARs.^9^

Finally, it is essential to consider the clinical significance of a dosimetric error. While for a given test case, it is possible to say whether the dosimetric change caused a dose constraint to be exceeded, this is highly dependent on the details of the individual dose distribution and may not generalize to other cases. Here, we detail a pragmatic approach for determining the likely clinical significance of dose differences across a patient cohort, with a view to prospective clinical use of the model.

## 2 MATERIALS AND METHODS

### 2.1 Dataset and clinical protocol

As this study was built based on previously published geometry study,^7^ you can find a summary of essential details information such as data preparation, OAR selection, gold standard contours, and image acquisitions in that earlier publication.^7^

A computer‐generated simple‐random list was used to select randomly 60 Brain cases from a retrospective clinical cohort treated in our institution over the past five years. Ethical approval for retrospective use of de‐identified patient data were given by Leeds East REC, reference: 19/YH/0300, IRAS project ID: 255 585. This UK ethics committee approval indicates that our study is conformant with the Declaration of Helsinki, the UK Policy Framework for Health and Social Care Research and the EMA guidelines on Good Clinical Practice. The data for training and testing was randomly chosen: 80% for training (*n* = 48) and 20% for testing (*n* = 12), which is the most popular split ratio (80/20).^7^ As the model used was a commercially approved model, on which we did not perform hyperparameter tuning, there was no need for in‐training validation. More information about the available training parameter can be found in the Supplementary Information.

Using the same dataset, two CT autosegmentation models were trained with a total of 47/48 cases (one case was excluded due to missing data), and three MRI autosegmentation models were trained using 32 cases (16/48 cases were excluded due to inconsistent MRI slice thickness).^7^ For testing, three test cases were excluded. Two CT test cases were excluded because no MRI images were associated with them (*n* = 10 cases) and one additional MRI test case was excluded (*n* = 9 cases) due to the use of different MRI sequence.^7^ In addition, All test cases were treated for either high‐grade or low‐grade glioma using volumetric modulated arc therapy (VMAT). Total RT dose was 60 Gy in 30 fractions, for glioblastoma multiforme (GBM) and grade III glioma (protocol A), or total RT dose was 54 Gy in 30 fractions for low‐grade glioma (protocol B). The clinical OAR dose constraints are shown in Table 1. D1, 5%, 50% denotes a minimum dose to the most exposed 1%, 5%, or 50% of the OAR volume, respectively.

**TABLE 1:** Dose constraints for glioma radical‐primary VMAT (60 Gy in 30# and 54 Gy in 30#).

OARs	Dose constrains	Dosimetric metrics
**Brainstem**	54 Gy	D5%
**Lenses**	6 Gy	D1%
**Optic chiasm**	54 Gy	D1%
**Optic nerves**	54 Gy	D1%
**Orbit**	45 Gy	D1%
**Lacrimal glands**	30 Gy	D1%
**Pituitary**	45 Gy	Max dose
**Cochlea**	45 Gy	D50%

### 2.2 Deep learning autosegmentation training

The OAR contours used for clinical treatment were based on a combination of the anatomy as seen on co‐registered MRI (specifically brainstem, optic chiasm, and intra‐cranial component of the optic nerves) and radiotherapy planning CT (specifically extra‐cranial portions of the optic nerves, lenses, globes, cochlea, and lacrimal glands). From these, the contours used in this project were derived:

1. Unedited clinical contours as above (used for both CT and MRI ‐based autosegmentation models, termed the CT unedited and MRI unedited models, CTu and MRIu, respectively‐ please see next paragraph)
2. Clinical contours edited to correspond with a departmental contouring guide (the “gold standard”) and edited to be entirely based on CT anatomy (used for CT and MRI ‐based autosegmentation models, termed the edited models CTeCT and MRIeCT‐ please see next paragraph)
3. Clinical contours edited to correspond with a departmental contouring guide and edited to be entirely based on MRI anatomy alone (used for the MRI‐based autosegmentation model termed the MRI edited model, MRIeMRI‐ please see next paragraph)

The same MRI and CT DL‐AC models that were built for geometric evaluation were used for dosimetric evaluation as follows:^7^

DL‐AC models were trained using a 3D U‐net^14^ architecture (RayStation 11A, RaySearch Laboratories AB, Stockholm, Sweden). Five separate autosegmentation models (two CT‐ and three MRI‐based) were trained: i) CT‐based, using the unedited clinical contours (CTu) and ii) CT‐based using contours edited to gold standard based on CT anatomy (CTeCT). Both contour sets were rigidly registered to T1‐weighted gadolinium‐enhanced MRI (T1w‐Gd MRI) to train the iii) MRI‐based model using the unedited clinical contours (MRIu), and the iv) MRI‐based model using the CT edited contours (MRIeCT). Finally, an MRI‐based model that used these contours edited based on MRI anatomy (MRIeMRI). After training, all the autosegmentation models were used to generate automatic contours on the test cohort.

### 2.3 Dosimetric evaluation

Dose statistics were computed (Raystation 11A) to compare the CT and MRI autosegmentation models with gold standard contours in each modality, where clinical contours were edited based on each modality's anatomy in this test cohort (i.e., CTeCT and MRIeMRI). Dose evaluation for MRI autosegmentation was performed by copying the CT dose distribution to T1w‐Gd MRI via rigid image registration.

The statistical significance of differences in dose metrics due to autosegmentation models was evaluated using a paired two‐tailed Student's *t*‐test. The three MRI‐based models were compared statistically, as were the two CT‐based models. The Bonferroni corrected statistical significance threshold was *p* ≤ 0.01 (0.05/3) and ≤ 0.05 for the MRI and CT dosimetric evaluations, respectively. *More information is available in the supplementary materials about dosimetric evaluation and statistical analysis*.

### 2.4 Clinical evaluation

The question of “what is a clinically significant dose difference?” is challenging. If an OAR dose is close to or at tolerance, any change could be significant, but we would normally accept a 2%−3% tolerance due to other uncertainties in dose calculation and setup (for example). However, that arbitrary 2%−3% tolerance would be overly restrictive if the OAR dose were said 30% below tolerance. Thus, the clinical significance of dosimetric differences for each OAR was determined using a pragmatic approach under the guidance of an experienced radiation oncologist.

**FIGURE 1:**
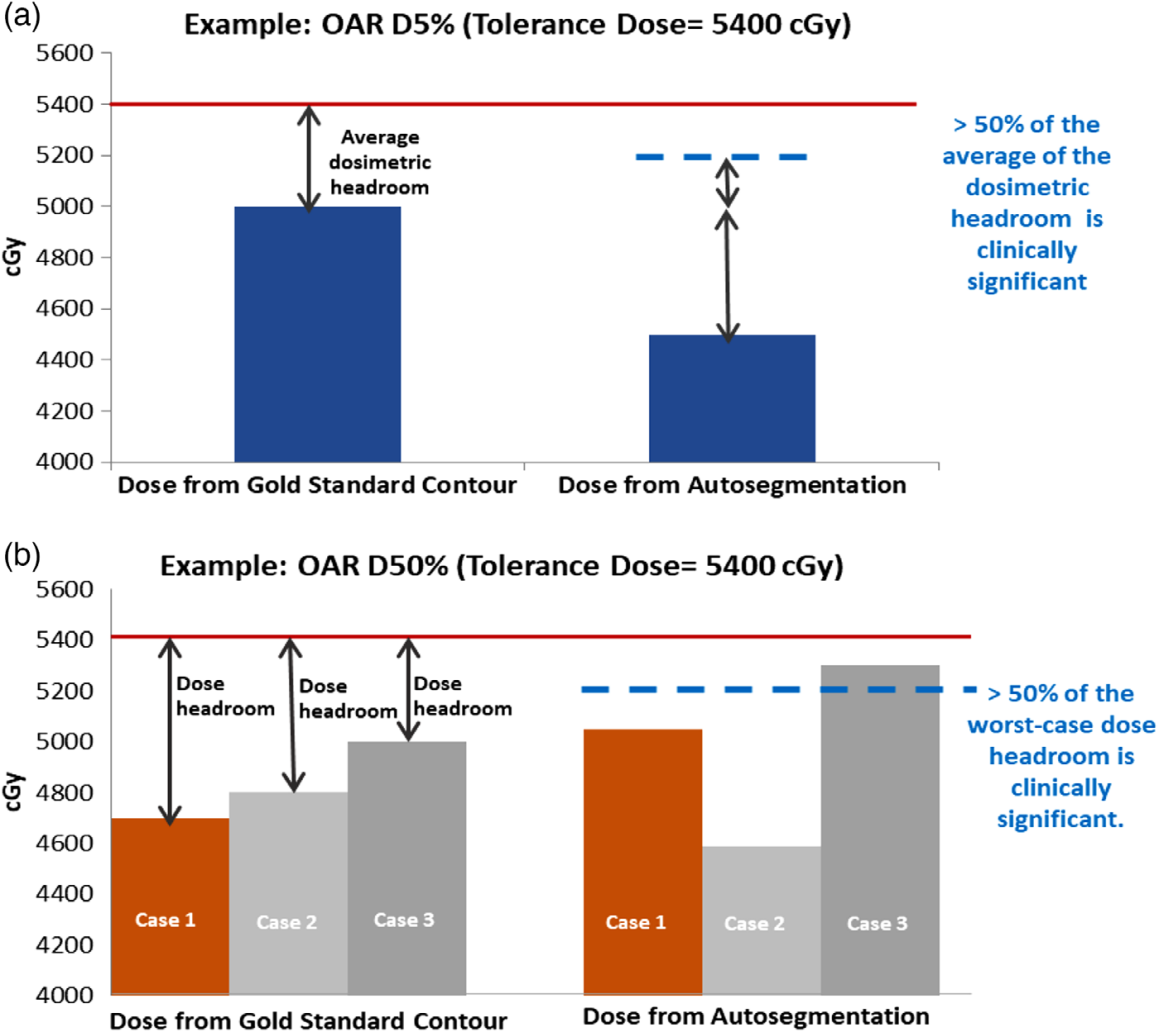
Clinical dose evaluation: (a) the average metric approach which relates to the average dose change, (b) the worst‐case scenario approach.

**For first‐order OARs** (where the dosimetric tolerance is a hard limit for RT dose planning) with near maximal dose statistics (e.g., D1% or D5%), the average dosimetric headroom between the gold standard contour dose and the tolerance dose in Table 1 was computed. 50% of the average dosimetric headroom was used as the clinical significance threshold for these first‐order OARs: brainstem, orbits, optic chiasm, and optic nerves. A case was considered clinically significant if the dose changes between the gold standard contour and autosegmentation was more than half the average dose headroom in either direction (Figure 1a).

**For second‐order OARs** (where dose tolerances are optimal, rather than mandatory) where the dosimetric statistics are mean‐dose‐like (e.g., cochlea D50%), the approach was based on the worst‐case scenario in the test cohort. The worst‐case scenario was defined as the case with the least headroom to the tolerance dose, using the gold standard OAR contours. 50% of the worst‐case scenario headroom was used as the clinical significance threshold. A case was considered clinically significant if the dose changes between gold standard contours and autosegmentation was more than half of this threshold in either direction (Figure 1b).

**For other second‐order OARs**, the clinical significance of the dosimetric change was more challenging to define. The evaluation was therefore based on a comparison of relative model dosimetric performance as above, rather than any clinical significance threshold. This approach was applied for lenses, lacrimal glands, and pituitary gland as they were treated in some cases to more than the optimal tolerance dose, which would result in a negative clinical significance threshold by the methods described above.

All cases identified as having clinically significant dosimetric changes were visually reviewed in the treatment planning system with an experienced clinical oncologist to identify the cause (e.g., proximity of an OAR to a dose gradient).

By aligning our approach with the perception of an experienced radiation oncologist, we enhanced the reliability of this clinically significant metric in identifying the potential clinically significant cases. As we mentioned in the introduction, there is no standard method or agreed threshold of acceptability for dosimetric variation.

### 2.5 Correlation between the geometric and dosimetric output

Pearson's Correlation Coefficient (*r*)^15^ was applied to measure correlations between geometric test metrics (the Dice Similarity Coefficient (DSC)^16^ sensitivity^8^ and mean distance to agreement (MDA)^17^ and absolute percentage dose change for each autosegmentation model.

## 3 RESULTS

### 3.1 Overall effect of using autosegmentation versus gold standard human contouring on dosimetry

**FIGURE 2:**
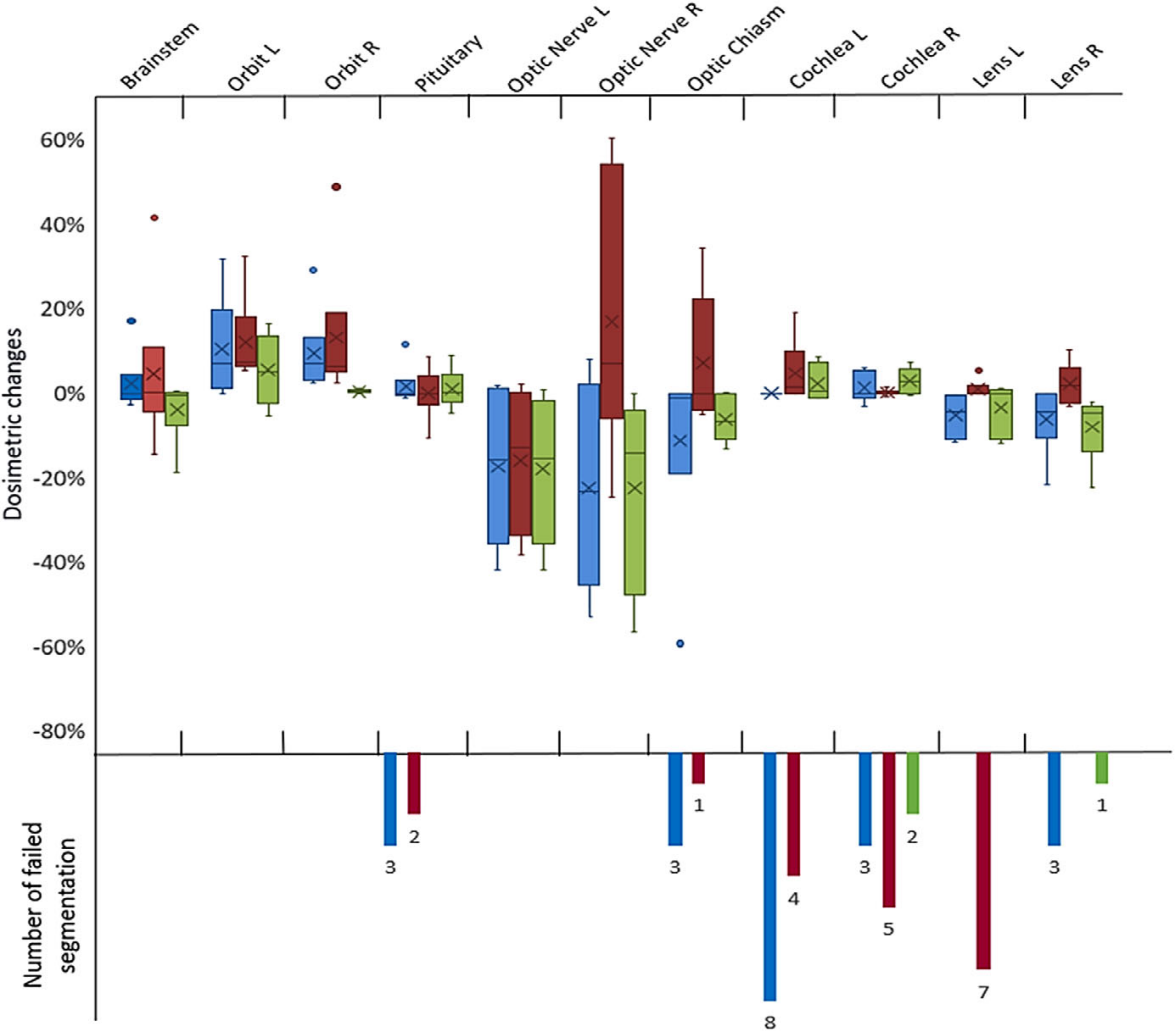
Distribution of the dosimetric change of all OARs delineated by MRI DL‐AC models (excluding lacrimal glands). The number of failed segmentations is when autosegmentation model failed to produce structures. In some cases, the small dosimetric change is affected by the number of failed cases such as cochlea, pituitary, and lens L. MRIu is shown in blue, MRIeCT in red, and MRIeMRI in green.

**FIGURE 3:**
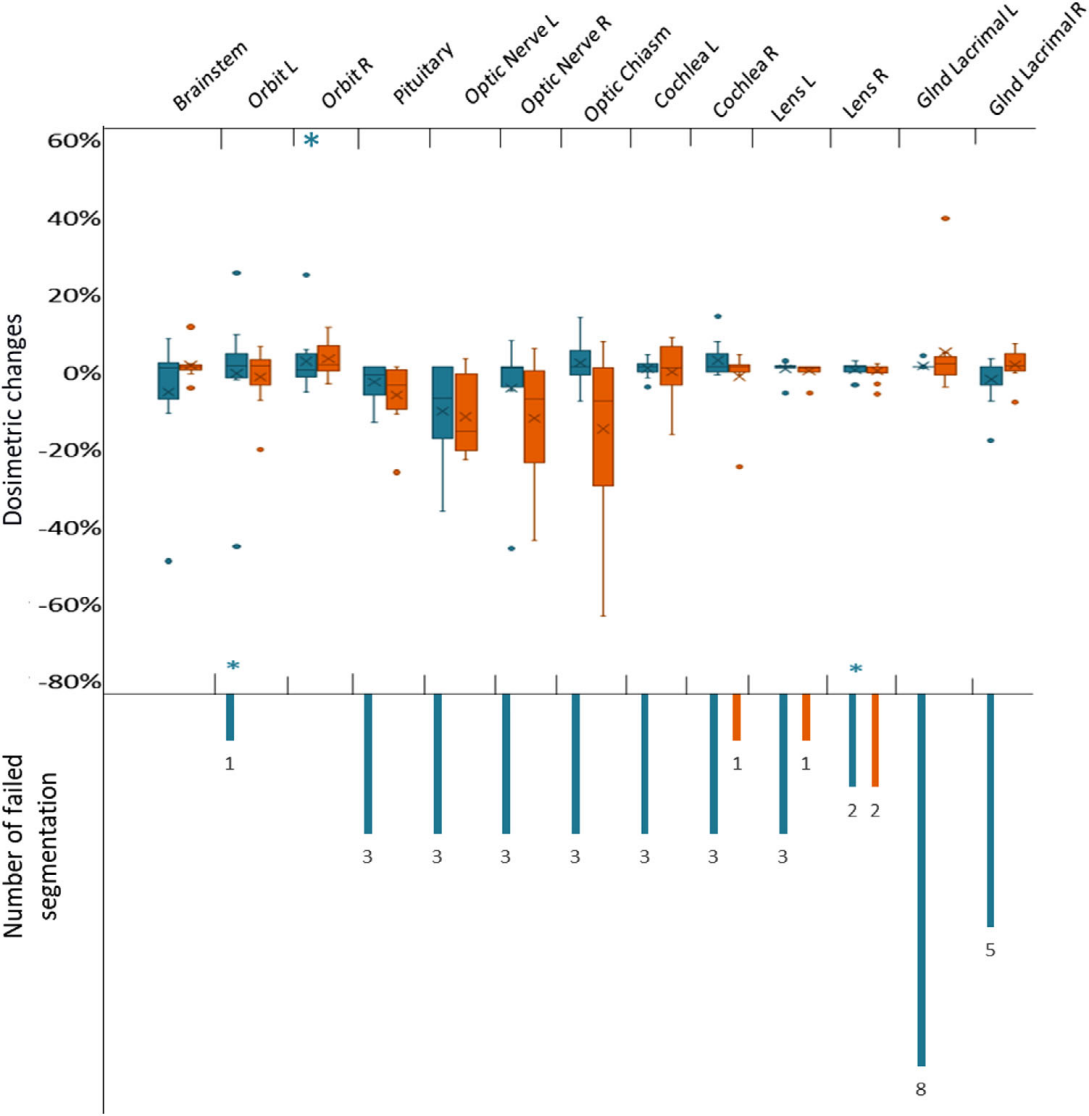
Distribution of the dosimetric changes of all OARs delineated by the CT DL‐AC relative to the gold standard contour. The number of failed segmentations is when autosegmentation model failed to produce structures. In some cases, the small dosimetric change is affected by the number of failed cases. CTu is shown in turquoise, while CTeCT is in orange. (*) indicates that outliers have been removed from the plot for clarity.

**FIGURE 4:**
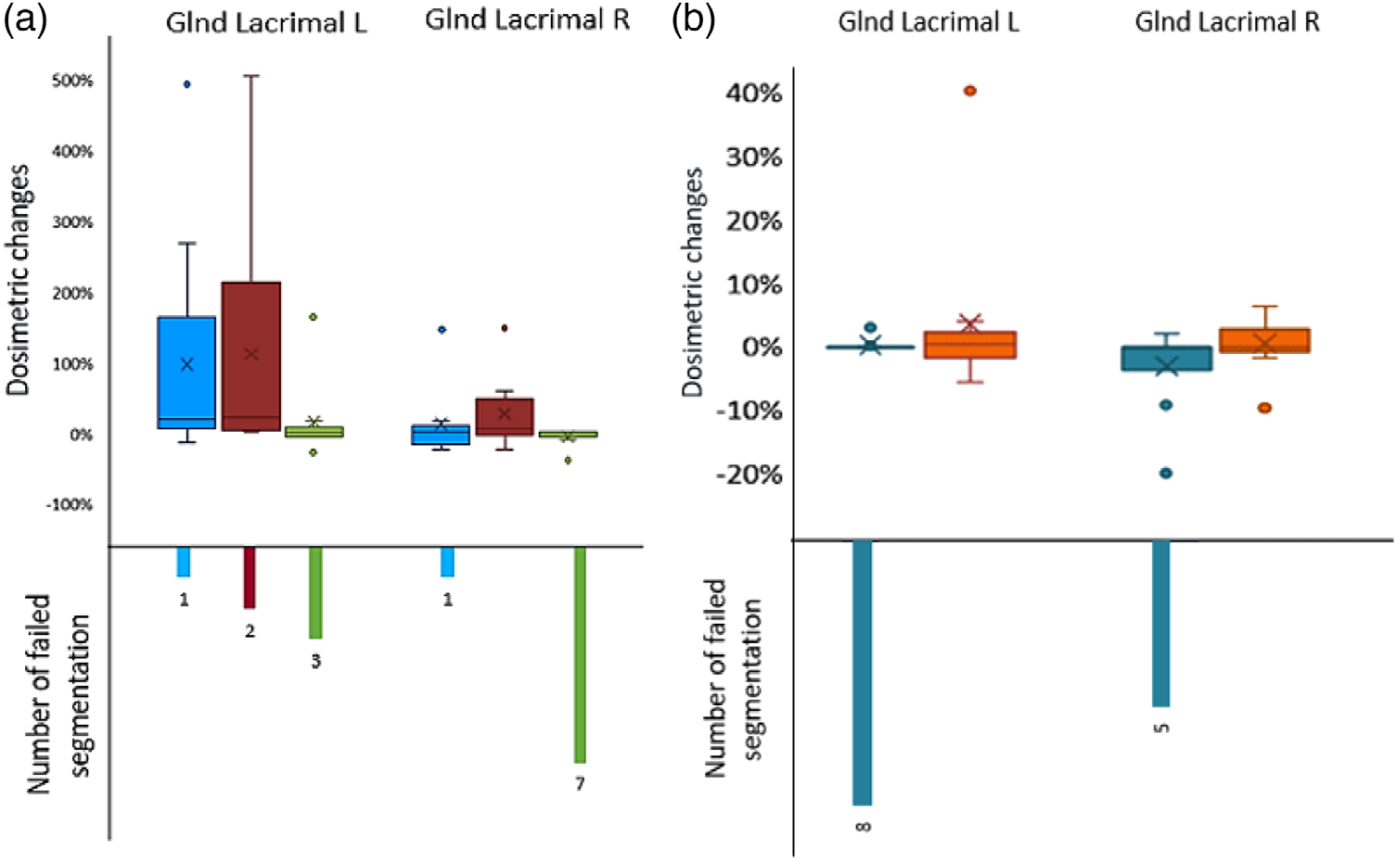
Distribution of the dosimetric change of the lacrimal glands segmented by (a) MRI DL‐AC models and (b) CT DL‐AC models relative to the gold standard contour. MRIu is shown in blue, MRIeCT in red, while MRIeMRI in green, CTu is shown in turquoise, while CTeCT in orange.

Figures 2, 3, 4 represent the overall patterns of dosimetric change for CT and MRI DL‐AC models relative to the gold standard contours. The lacrimal glands are presented separately due to the relatively larger dose changes. The dosimetric change for the MRI autosegmentations versus gold standard contour was greatest in the lacrimal glands D1%, followed by the optic nerves D1% (Table 2) (Figures 2 and 4). The average absolute dosimetric change for the lacrimal glands D1% and optic nerves D1% varied from 25% (MRIeMRI) to 143% (MRIeCT) and 9% (MRIeMRI) to 20% (MRIu and MRIeCT), respectively (Table 2). The remaining OARs had less dosimetric change relative to the gold standard contour, ranging from 1% to 12% (Table 2).

**TABLE 2:** The absolute average dosimetric change between the MRI autosegmentations and gold standard contour.

	Brainstem D5%	Cochlea L D50%	Cochlea R D50%	Lacrimal L D1%	Lacrimal R D1%	Lens L D1%	Lens R D1%	Optic Chiasm D1%	Optic Nrv L D1%	Optic Nrv R D1%	Orbit L D1%	Orbit R D1%	Pituitary max dose
**MRIu**													
**Δ absolute average dosimetric change**	3%	1%	3%	113%	29%	4%	7%	12%	20%	20%	9%	10%	3%
N*	9	1	6	8	8	9	6	6	9	9	9	9	6
**MRIeCT**													
**Δ absolute average dosimetric change**	7%	6%	1%	143%	33%	3%	4%	9%	15%	20%	10%	11%	4%
N*	9	5	4	7	9	2	9	8	9	9	9	9	7
**MRIeMRI**													
**Δ absolute average dosimetric change**	3%	3%	10%	36%	25%	3%	7%	4%	9%	18%	6%	1%	3%
N*	9	9	7	6	2	9	8	9	9	9	9	9	9

*Note*: N* represents the number of the successful segmentation by each model.

The greatest dosimetric change for the CT DL‐AC versus gold standard contour was observed for the right lens D1% and optic chiasm D1% for the CTu and CTeCT models, respectively (Table 3 and Figure 3). The average absolute dosimetric change was 57% (CTu) for the right lens D1% and 18% (CTeCT) for the optic chiasm D1%. The marked dosimetric change was also reported for L and R orbits D1% delineated by the CTu model (21%, 25%) and L and R optic nerves D1% delineated by the CTeCT model (14%, 15%) (Table 3). The remaining OARs had less dosimetric change relative to the gold standard contour, ranging from 1% to 17% (Table 3).

**TABLE 3:** The absolute average dosimetric change between the CT autosegmentations and gold standard contour.

	Brainstem D5%	Cochlea L D50%	Cochlea R D50%	Lacrimal L D1%	Lacrimal R D1%	Lens L D1%	Lens R D1%	Optic Chiasm D1%	Optic Nrv L D1%	Optic Nrv R D1%	Orbit L D1%	Orbit R D1%	Pituitary max dose
**CTu**													
**Δ absolute average dosimetric change**	9%	2%	4%	2%	8%	2%	57%	6%	17%	10%	21%	25%	6%
N*	10	7	7	8	5	7	8	7	7	7	9	10	7
**CTeCT**													
**Δ absolute Average dosimetric change**	2%	5%	4%	6%	3%	1%	2%	18%	14%	15%	5%	4%	8%
N*	10	10	9	10	10	9	8	10	10	10	10	10	10

*Note*: N* represents the number of the successful segmentation by each model.

### 3.2 Impact of editing

For orbits, optic nerves, and optic chiasm, the MRIeMRI model showed less average dosimetric changes than other MRI models (Table 2 and Figure 2). However, differences between MRI DL‐AC model dosimetry were not statistically significant, except in the right orbit, where a statistically significant effect was found comparing the MRIu and MRIeMRI models (*p *= 0.012, effect size (Δ median dosimetric change) = 7%). However, it was clinically insignificant (Table S1).

The CTeCT model demonstrated smaller average dosimetric changes, relative to the gold standard, than the CTu model for the following structures: orbits, lenses, and brainstem (Table 3 and Figure 3). Again, however, dosimetric differences between the CT DL‐AC models were not statistically significant (Table S2). In 3 cases, the CTu model generated incorrectly located segmentations (DSC = 0) for several of these OARs. These cases were visually qualitatively assessed in the treatment planning system.

### 3.3 MRI versus CT DL‐AC—effect on dosimetry

Differences in dosimetric changes relative to the gold standard contour between the CT and MRI DL‐AC models, were most noticeable in the lacrimal glands D1% (Figure 4). The dosimetric change for lacrimal glands D1% delineated by the CT DL‐AC models versus gold standard contour was considerably smaller than that of the MRI DL‐AC models. Additionally, the MRIeMRI model failed to segment the lacrimal glands in nine cases.

### 3.4 Correlation between the geometric and dosimetric evaluations

All models showed a weak correlation between absolute dosimetric change and geometric evaluation metrics. Negative correlations were observed between DSC and absolute dosimetric change and between sensitivity and absolute dosimetric change (*r* ≤ −0.40 and *r* ≤ −0.38, respectively). A positive correlation was observed between mean DTA and absolute dosimetric change (*r* ≤ 0.54) (Table 4). None of the observed correlations reached statistical significance at *p* = 0.05. *All results related to the geometric output used for this evaluation can be found in the previous published work*.^7^

**TABLE 4:** Correlation between geometric and dosimetric outputs.

Autosegmentation models	Absolute dosimetric change and DSC	Absolute dosimetric change and sensitivity	Absolute dosimetric change and mean DTA
**MRIeCT**	*r* = −0.299	*r* = −0.256	*r* = 0.262
**MRIeMRI**	*r* = −0.402	*r* = −0.381	*r* = 0.328
**MRIu**	*r* = −0.304	*r* = −0.255	*r* = 0.543
**CTeCT**	*r *= −0.343	*r* = −0.378	*r* = 0.288
**CTu**	*r* = −0.386	*r* = −0.359	*r* = 0.106

### 3.5 Clinical significance of autosegmentation models on dosimetry

#### 3.5.1 First‐order OARs

Tables 5 and 6 demonstrate the number of clinically significant cases according to the definitions outlined above, and the average dosimetric change relative to the gold standard contour. In both CT DL‐AC and MRI DL‐AC, the number of cases that exceeded the clinical significance threshold for optic chiasm D1% was higher than for other first‐order OARs (*n* ≥ 4 cases). In both modalities, models trained with edited contours based on CT scans (MRIeCT and CTeCT) demonstrated the largest frequency of clinically significant errors (*n* = 7 with Δ average dose = 590.0 and 1376.1 cGy, respectively) (Table 6).

**TABLE 5:** Significant clinical cases and their average of the dosimetric change compared to gold standard.

	Threshold (cGy)		MRIeCT		MRIeMRI		MRIu	
OARs	Protocol A	Protocol B	A (*n* = 5 cases)	B (*n* = 4 cases)	A (*n* = 5 cases)	B (*n* = 4cases)	A (*n* = 5 cases)	B (*n* = 4 cases)
**Brainstem D5%**	54.222	660.410	(*n* = 1) 411.3 cGy	–	(*n* = 1) 58.9 cGy	–	(*n* = 1) 140.3cGy	–
**Cochlea L D50%**	548.633	1001.294	(*n* = 1) 636.4 cGy	–	–	–	–	–
**Cochlea R D50%**	42.351	380.431	–	–	(*n* = 1) 1341.498 cGy	–	(n = 1) 54.5 cGy	–
**Optic Chiasm D1%**	50.231	286.879	(*n* = 5) 164.8 cGy	(*n* = 2) 1015.2 cGy	(*n* = 3) 298.1 cGy	(*n* = 1) 722.0 cGy	(*n* = 3) 160 cGy	(*n* = 1) 1745.9 cGy
**Optic Nrv L D1%**	521.292	1031.981	(*n* = 1) 869.8 cGy	–	(n = 2) 611.3 cGy	–	(*n* = 2) 1269.393 cGy	(*n* = 1) 1192 cGy
**OpticNrv R D1%**	1194.213	1063.127	–	(*n* = 1) 1313.7 cGy	–	(*n* = 1) 1123.8 cGy	–	(*n* = 1) 1073.1 cGy
**Total clinically significant cases**			**11**		**9**		**10**	

**TABLE 6:** Significant clinical cases and their average of the dosimetric change compared to the gold standard.

	Threshold (cGy)		CTu		CTeCT	
OARs	Protocol A	Protocol B	A (*n* = 7 cases)	B (*n* = 3 cases)	A (*n* = 7 cases)	B (*n* = 3 cases)
**Brainstem D5%**	171.161	43.310	(*n* = 3) 447.9 cGy	(*n* = 2) 279.4 cGy	(*n* = 2) 395.8 cGy	(*n* = 1) 97.3 cGy
**Cochlea L D50%**	287.110	79.972	–	–	(*n* = 1) 747.9 cGy	–
**Cochlea R D50%**	340.995	394.289	–	–	(*n* = 1) 668.8 cGy	–
**Optic Chiasm D1%**	186.969	161.208	(*n* = 3) 402.527 cGy	(*n* = 1) 503.8 cGy	(*n* = 4) 825.6 cGy	(*n* = 3) 1926.5cGy
**Optic Nrv L D1%**	769.175	821.592	(*n* = 1) 768 cGy	(*n* = 1) 1605.9 cGy	(*n* = 2) 996.2 cGy	(*n* = 1) 1025.7cGy
**Optic Nrv R D1%**	1092.199	561.628	(*n* = 1) 1226.1 cGy	–	(*n* = 1) 1173.0 cGy	–
**Orbit L D1%**	1806.533	2038.483	(*n* = 1) 2572.2 cGy	–	–	–
**Total clinically significant cases**			**13**		**16**	

Only one clinically significant case was observed for the brainstem D5% in each MRI DL‐AC model (*n *= 3 cases, Δ average dose = 203.5 cGy) (Table 5). However, the MRIeCT exhibited greater dosimetric change relative to the gold standard contour than the MRIeMRI and MRIu models (Table 5).

Training the CT DL‐AC model with edited contours, on the other hand, reduced the frequency of clinically significant dosimetric errors for the brainstem D5% and demonstrated smaller dosimetric changes relative to the gold standard contour compared to the CTu model (*n* = 3 cases, Δ average = 246.6 cGy) (Table 6).

#### 3.5.2 Second‐order OARs

Amongst the second‐order OARs (waterfall plots—Figures S1 and S2), the lacrimal glands demonstrated the largest dosimetric change in the MRI DL‐AC models. In the worst case, the dose was changed in the left lacrimal gland by 505% for the MRIeCT model (Figure S1c), relative to the gold standard. On the other hand, the right lens had the largest dosimetric change in the CT DL‐AC (446% worst‐case for the CTu model) (Figure S2b). Otherwise, the dosimetric changes associated with DL‐AC compared to gold standard were generally lower for second‐order OARs. For CT DL‐AC, these ranged from 0% to 40%, whereas they ranged from 0% to 22% for the MRI DL‐AC (Figure S1(a–e) and S2(a‐e)).

## 4 DISCUSSION

This study investigated the dosimetric impact of clinical contour editing before training MRI and CT DL‐AC models for brain OARs to establish clinical applicability. This study also examined the correlation between geometric and dosimetric outcomes, in order to guide centers as to whether the geometric assessment alone is sufficient to evaluate and commission DL‐AC models in radiotherapy or whether a dosimetric evaluation is also necessary.

Except for the right orbit, when delineated by the MRI models, the dosimetric statistical analysis revealed no superior model between the CT DL‐AC models or between the MRI DL‐AC in terms of the dosimetric accuracy for any investigated brain OARs (Tables S1 and S2). The significant finding for the right orbit likely results from a slight registration inaccuracy in mapping CT‐derived evaluation contours to MRI, rather than any feature of the DL‐AC model. As a result, editing contours for brain OAR structures on the CT or MRI scans before training the model had no significant effect on OAR dosimetry. The lack of superiority indicates that both models perform well dosimetrically. This occurs for two main reasons. Firstly, doses in brain RT for GBM are relatively homogeneous, meaning that most differences between these complex OAR contours lie in either uniformly high or low dose regions. Only occasionally will a contouring difference occur on a high dose gradient, leading to a significant dosimetric impact. Secondly, the metrics used clinically tend to be of the “near‐maximal dose” type, which are insensitive to contouring changes which occur in regions of lower dose. This is in contrast to metrics such as mean doses or V20Gy, which might be used in the thorax for example.

Clinical dosimetric evaluation was performed as a secondary assessment of potential clinical impact, using the average metric approach and the worst‐case scenario approach.

The number of patients that exceeded the derived clinical significance threshold for optic chiasm D1% was higher compared to other OARs (brainstem D5%, orbits D1%, optic nerves D1%, and cochlea D50%) in both modalities.

The absolute dosimetric changes of the optic chiasm D1% relative to the gold standard of the clinically significant cases were ≤67% and ≤59% across all the CT and MRI models, respectively. The DSC, sensitivity and mean DTA scores were ≤0.37, 0.54, 1.44 cm and ≤0.74, 0.83, 0.77 cm for the CT and MRI models, respectively.^7^ In comparison with the CTeCT model, the CTu model shows a smaller number of significant cases with more acceptable percentage change, which is surprising at first sight since the edited contours should be more closely correlated with the underlying CT anatomy. However, as the optic chiasm is very poorly visualized on CT, the segmentation model relies not on the correlation with imaging features, but more on the consistency of the shape and location of the optic chiasm, to predict its segmentation. In the unedited data (used for the CTu model), this consistency is high, due to the original clinical contours being based on MRI rather than CT anatomy (see Section 2.2), enabling the model to learn. By editing the optic chiasm on CT anatomy alone (used for the CTeCT model), this consistency is degraded, and the correlation with image features is not improved, as there are none present on CT. Hence, CTeCT performs worse, as it struggled to learn a consistent shape and location for the optic chiasm and hence produced a high dosimetric change with more significant cases compared to CTu.

On the other hand, MRIeMRI showed a more acceptable dosimetric change than other MRI models, showing the benefits of editing optic chiasm on MRI prior to model training. Based on visual inspection of the optic chiasm in the treatment planning system, the level of dose discrepancy was independent of dose gradient location and appeared well correlated to the geometric error. This is expected because optic chiasm is a small structure and has a complicated shape. The model failed to delineate all the optic chiasm on each slice accurately. This indicates that post‐segmentation editing may be required for optic chiasm.

Regarding the Brainstem D5%, in a comparison with the CTu model, the CTeCT model demonstrated a lower number of significant changes for brainstem D5% (3 significant cases) (Table 6) with less dosimetric change relative to the gold standard (≤11% in either direction). Notably, the CTu model segmented several OARs in completely the wrong location, leading to the significant increase in mean dosimetric errors for the brainstem (and orbits). Editing prior to model training resolved these failures.

On the other hand, the number of clinically significant cases for the brainstem D5% for the MRI was just one for each model (Table 5). The dosimetric differences compared to the gold standard contour were ≤19% in either direction, but the geometric error was low (DSC and sensitivity scores ≥0.89 and 0.85, while mean DTA score ≤0.10 cm).^7^ This dosimetric error appears clinically significant because in most clinical cases, D5% Brainstem is at or near to the PTV, so even a slight difference is significant.

**FIGURE 5:**
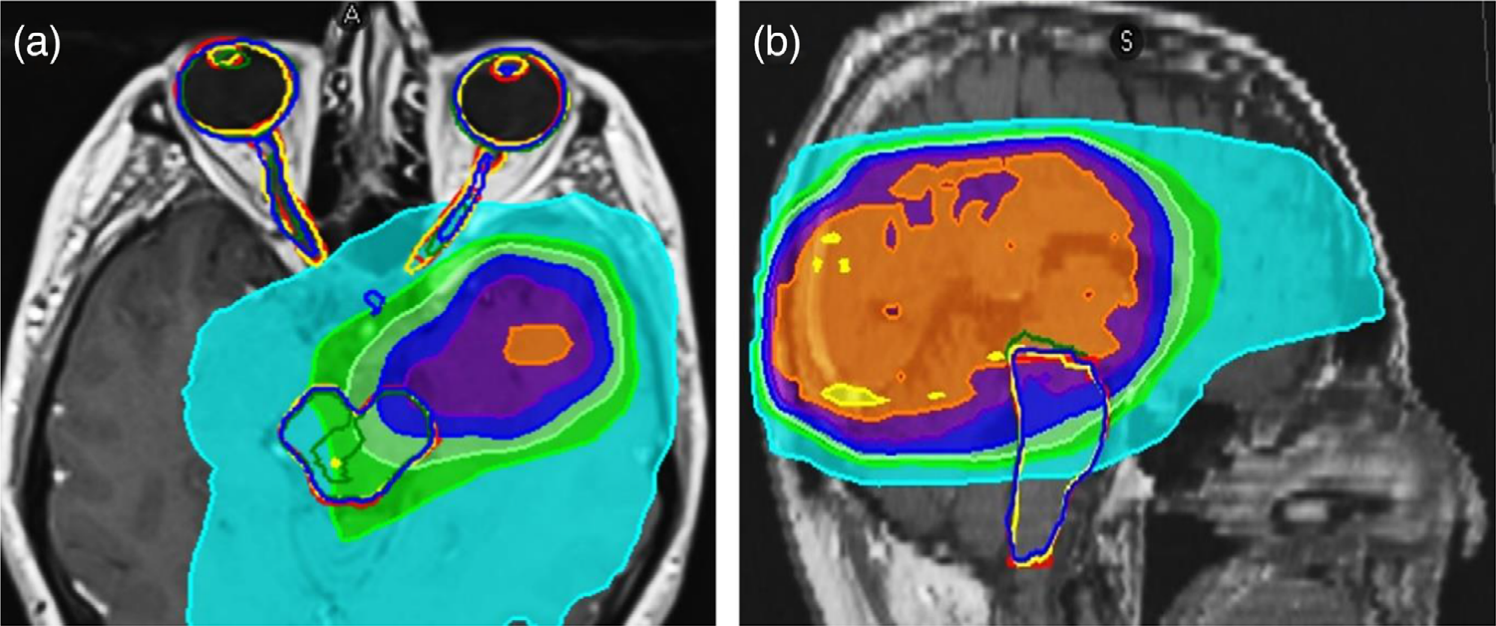
(a) axial and (b) sagittal T1w‐Gd MRI with overlying dose distribution, showing examples of different geometrical changes of predicted MRI autosegmentations compared to the gold standard. Red outline represents the gold standard contour. The MRIeMRI contours are depicted as yellow outlines, the CTeMRI contours as green outlines, and MRIu contours as blue outlines. The colorwash represents the percentage dose distribution, relative the prescription dose, according to the inset colorbar. The dosimetric impact for a given geometric error is large only in high‐dose gradients (e.g., as seen on sagittal image, the dosimetric impact of the yellow contour, relative to the gold standard (red) is 7% (411 cGy), as there is a steep dose gradient, whereas the dosimetric difference of the green contour relative to gold standard (red) is only 1% (38 cGy), as it lies in a more homogenous region of dose). Overall, this dependence on dose gradient leads to the observed weak overall correlation of dosimetric impact and geometric error.

It was noticeable that the geometric error of the clinically significant cases for D5% brainstem in both modalities was generally low (DSC score ≥0.8),^7^ except for CTu failure cases mentioned above. On visual assessment, the superior part of the brainstem was found to overlap PTV or at a distance, resulting in significant dose gradients (Figure 5a). These results show that clinical dosimetric evaluation is essential in some cases, and the geometric evaluation alone is insufficient to demonstrate the clinical utility of autosegmentation, due to the extreme inhomogeneity of dose distributions. Geometric errors only translate to dosimetric errors where they overlap steep dose gradients. Similarly, a recent study evaluating the dose for the thoracic OARs delineated by CNN‐based autosegmentation found that significant dose‐volume variations were more strongly correlated with areas of high‐dose gradient than geometric segmentation errors.^6^ Moreover, previous studies have identified significant dosimetric differences between test and standard segmentation observed for OARs with high‐dose gradients, even when geometric measures show good overlap.^4^ On the other hand, OARs within homogeneous dose regions may reveal poor volumetric agreement but minimal dosimetric differences.^4^ Accordingly, the superior part of the brainstem autosegmentation must be corrected when needed due to the poor performance of the contouring models in this portion and due to its proximity to PTV in many of the GBM cases (Figure 5a). For modern, highly conformal arc therapies, the PTV is often a good surrogate for the location of high‐dose gradients, but care should be taken with fixed beam angle treatments, where steep gradients may exist far from the PTV.

It was noted that clinically significant dosimetric changes for optic nerves were mostly reductions in dose relative to the gold standard for two main reasons. First, after reviewing the treatment planning system, the CT models were found to have failed to correctly identify all the boundaries on each slice, while the MRI models failed to identify the posterior limit of the optic nerves (Figure 5b). In either case, the segmentation was incomplete, resulting in reduced dose statistics. Second, a considerable reduction in dose was noticed in some other cases, even though there was relatively good visual agreement between the generated contours and the gold standard. In these cases, part of the gold standard contour was near PTV, whereas the DL‐AC contour was not.

Regarding the small structures (lenses, lacrimal glands, and pituitary), lacrimal glands delineated by MRI models demonstrated a remarkably high dosimetric change relative to the gold standard. This correlated with geometric inaccuracy due to the difficulty visualizing this organ using T1‐w MRI scans.^7^ Uniquely, on CT, these glands are more visible than on MRI. However, CTu model showed that the right lens had the largest dosimetric change (446% worst‐case). This was a failed segmentation, which falsely identified a region of the brain far from the right lens, instead of simply producing no contour. The dose in that region was approximately 4.5× higher than in the lens, as it was by chance on the PTV boundary, leading to this extreme result.

This study found a weak correlation between the geometric and dosimetric outcomes in both modalities. The correlation direction of the geometric and dosimetric results followed our expectations. The absolute percentage dose difference was negatively correlated with the sensitivity and DSC scores, and positively correlated with the mean DTA. Higher DSC and sensitivity scores indicate improved geometric performance, whereas higher mean DTA scores indicate larger geometric (and hence dosimetric) errors.

This suggests geometric test metrics were insufficient to predict the effect of contour inaccuracies on dose, due primarily to variability in the location of dose gradients. Also, geometry test metrics such as DSC can be impacted by the structure size and are often a poor indicator of clinically significant dosimetric impact.^3^, ^4^

A recent study examined the correlation between geometrical measures and dose‐volume variations for thoracic OARs.^6^ Researchers found no significant correlation between them.^6^ The weak correlation identified in this current work may indicate that dose distributions exhibit more variance in the thorax than the brain; hence, geometric performance was found to be an insufficient metric for clinical utility. Consequently, it is crucial also to perform dosimetric tests to demonstrate the clinical applicability and accuracy of autosegmentation models.

The fact that specific organs are prone to exhibiting large geometric errors, and the likelihood that these are in high‐dose gradient regions, potentially allows human operators to prioritize their contour editing to the critical organs that are likely to be in the vicinity of high‐dosegradients, further improving efficiency in checking contours, and avoiding spending time editing geometric errors which will not translate to dosimetric errors.

This study has certain limitations. The relatively small number of cases analyzed makes it possible that outlier cases have not been captured (e.g., where a small OAR lies very close to a high‐dose gradient). Additionally, the clinician's time editing contours needs to be investigated to measure efficiency savings from DL‐AC.

## 5 CONCLUSION

As technology advances and the number of brain cancer patients increases, clinical use of brain OARs DL‐AC models in the radiotherapy department becomes attractive. However, adequate assessment of contour accuracy and clinical applicability are essential. In this study, the dosimetric impact of autocontouring OARs in the brain was investigated. Specifically, the dosimetric impact of editing clinical contours to gold standard quality before training CT and MRI DL‐AC models was assessed. Moreover, the correlation of dosimetry with geometric accuracy of MRI and CT‐based DL‐AC models was determined. Generally, we found that editing the clinical contour before training the model had no statistically significant impact on the dosimetry, despite clear geometric effects. However, by assessing the clinical significance of dosimetric changes as a secondary assessment of potential clinical impact, some geometric errors resulted in clinically significant dosimetry changes, despite the small underlying geometrical errors.

Our results suggest that an MRIeMRI model could be used clinically for treatment planning despite some structures requiring manual contour editing. This is due to its generated segmentation generally showing less dosimetric change relative to the gold standard contours for most of the OARs. It also produced the fewest clinically significant dosimetric errors, indicating that the improvements in geometric performance can lead to dosimetric improvements in specific cases.

Generally, a weak, and statistically insignificant correlation between the geometric and dosimetric outcomes for brain OARs in both modalities was found. Accordingly, geometric test metrics are insufficient to establish the impact of autocontouring inaccuracies on RT dose, mainly due to the variability in the location of dose gradients relative to OARs and geometric errors. For robust evaluation and commissioning of autocontouring, both geometric and dosimetric evaluation is recommended.

## AUTHOR CONTRIBUTIONS

Nouf Alzahrani was responsible for collecting, preparing, and analyzing the data, model training and testing, interpreting the results, and writing the manuscript. Michael Nix and Louise Murray provided essential guidance for the study design, reviewing the analysis and interpretation of the result. Ann Henry and Bashar Al‐Qaisieh contributed to reviewing and approving the study design from the clinical and technical perspectives and providing the overall guidance of the project and data. Anna Clark supported in extracting the data from the treatment planning system. All authors contributed to the review of the manuscript, and all approved the final draft for submission.

## CONFLICT OF INTEREST STATEMENT

The authors declare no conflict of interest.

## Supporting information

Supplemental Information.

Supplemental Information.

Supplemental Information.
