# Supplementary material for: Dosimetric impact of contour editing on CT and MRI deep‐learning autosegmentation for brain OARs
Source: J Appl Clin Med Phys. 2024 Apr 25;25(5):e14345. doi: 10.1002/acm2.14345 (PMC11087158; doi:10.1002/acm2.14345)
Supplement: Supplementary file 1 — Supplemental Information. [file ACM2-25-e14345-s001.pdf]

Supplementary Information

- ***Figures***

Figure 1S and 2S:

Waterfall plots illustrated the domsimetric change between the gold standard contour and autosegmentation models. MRIu is shown in blue, MRIeCT in red, while MRIeMRI in green, CTu is shown in turquoise, while CTeCT in orange. Note: Stars (*) for the cases were not segmented by the model in all the following graphs.

Figure 1S (a) to (e):

Figure 1S: a: Perecentage Dose change between the Gold Standard contour and MRI autosegmentation models for the Lens_L D1%

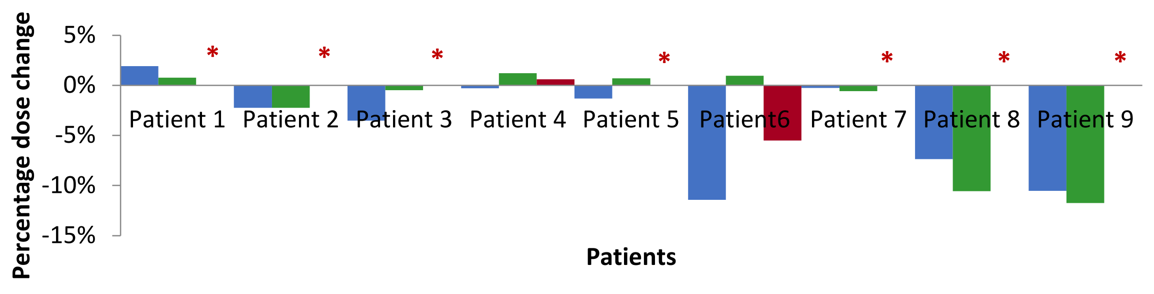

Figure 1S: b) Perecentage Dose change between the Gold Standard contour and MRI autosegmentation models for the Lens_R D1%

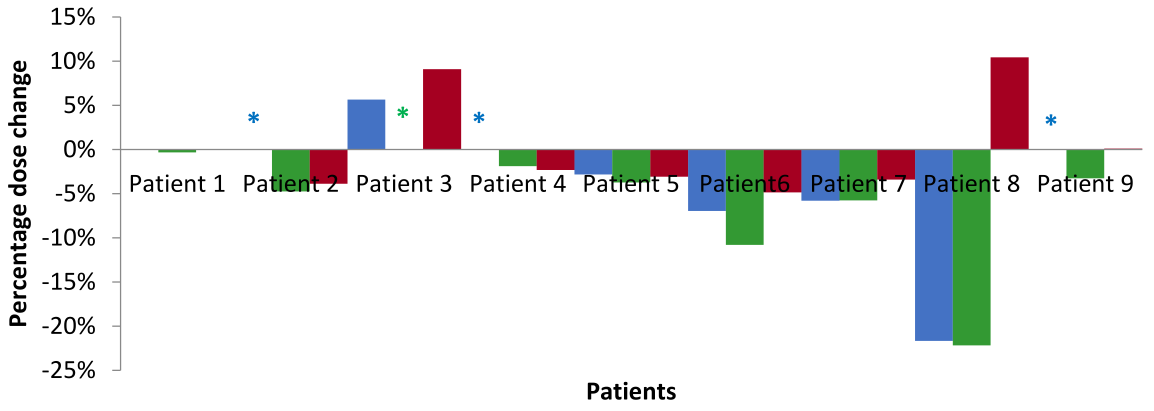

Figure 1S: c) Perecentage Dose change between the Gold Standard contour and MRI autosegmentation models for the Glnd_Lacrimal_L D1%

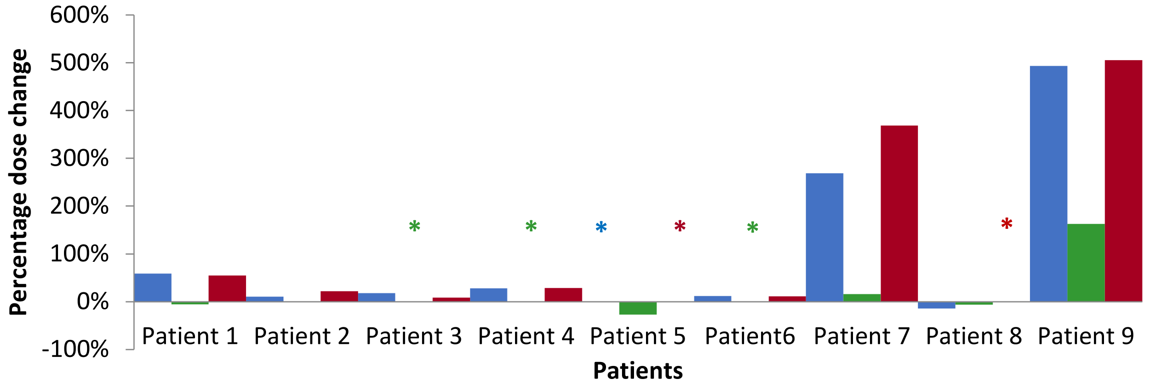

Figure 1S: d) Perecentage Dose change between the Gold Standard contour and MRI autosegmentation models for the Glnd_Lacrimal_R D1%

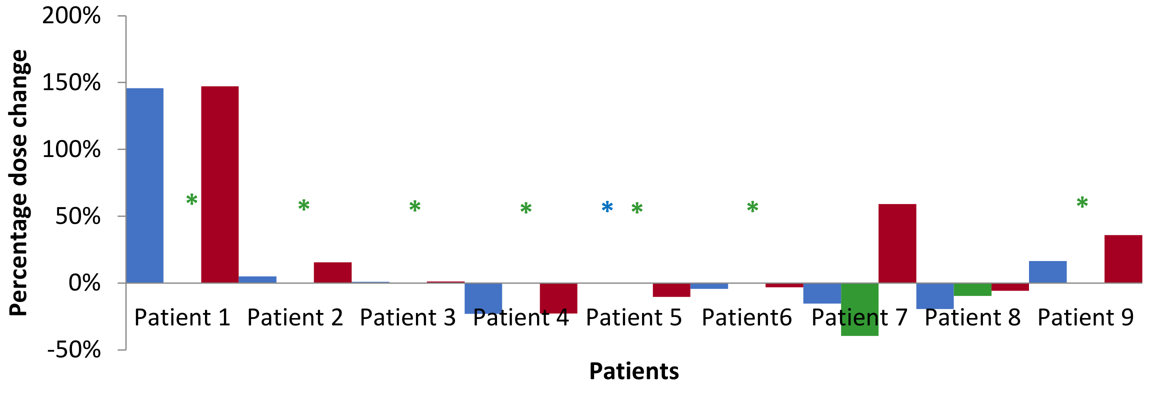

Figure 1S:e):Perecentage Dose change between the Gold Standard contour and MRI autosegmentation models for the Pituitary max dose (cGy)

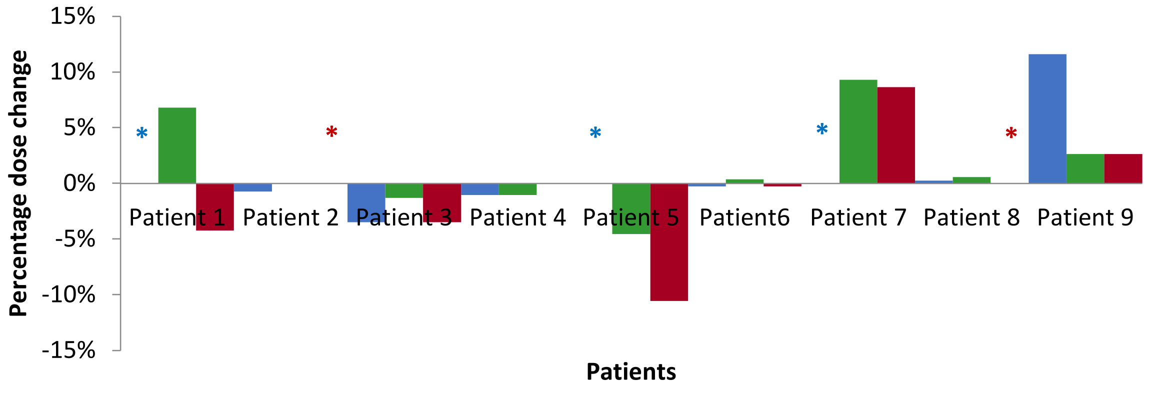

Figure 2S (a) to (e):

Figure 2S:a) Perecentage Dose change between the Gold Standard contour and CT autosegmentation models for the Lens_L D1%

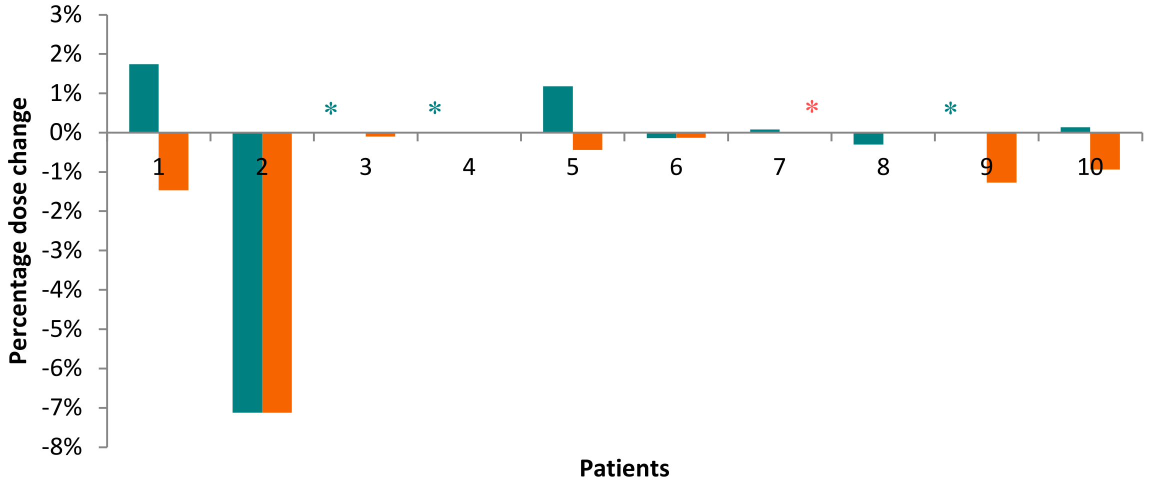

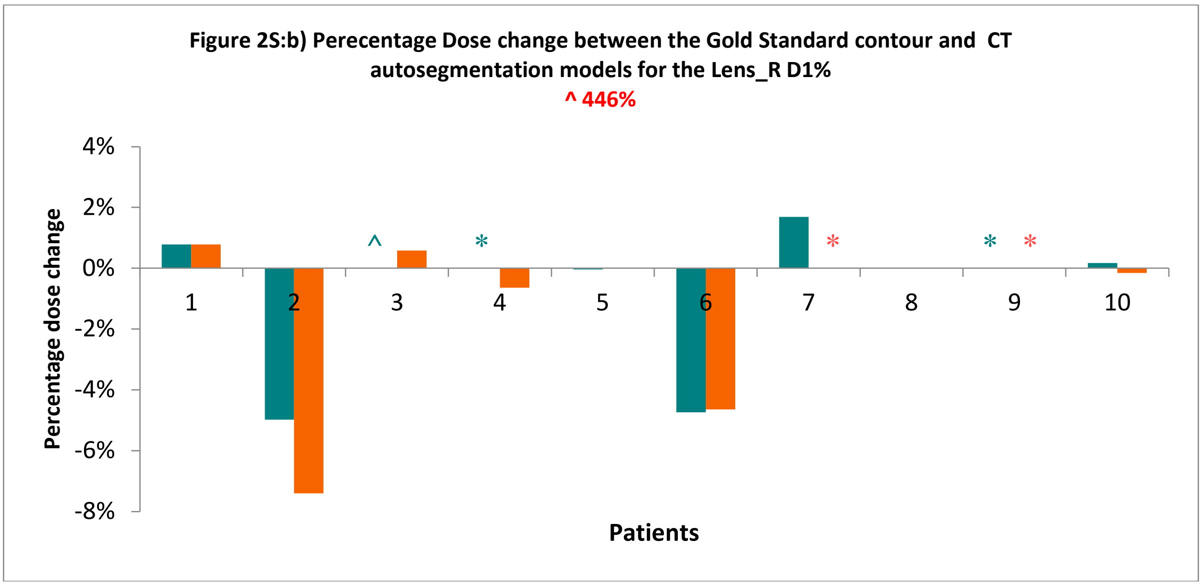
Figure 2S:b) Perecentage Dose change between the Gold Standard contour and CT autosegmentation models for the Lens_R D1%
^ 446%
4%
2%
0%
-2%
-4%
-6%
-8%
Percentage dose change
^
*
*
*
*
1
2
3
4
5
6
7
8
9
10
Patients

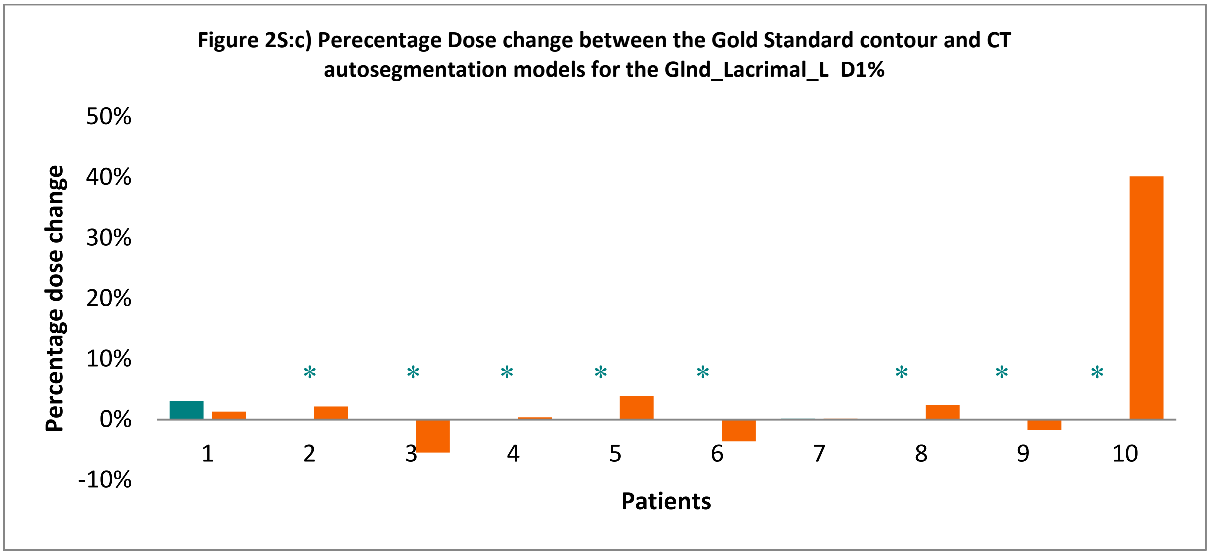
Figure 2S:c) Perecentage Dose change between the Gold Standard contour and CT autosegmentation models for the Glnd_Lacrimal_L D1%
50%
40%
30%
20%
10%
0%
-10%
Percentage dose change
*
*
*
*
*
*
*
*
1
2
3
4
5
6
7
8
9
10
Patients

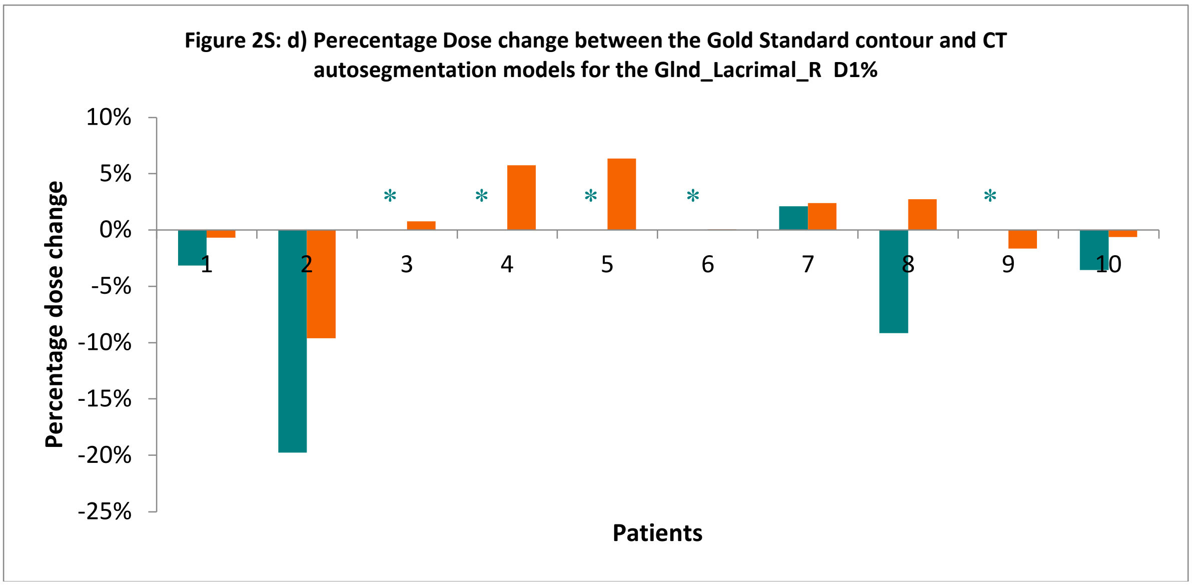
Figure 2S: d) Perecentage Dose change between the Gold Standard contour and CT autosegmentation models for the Glnd_Lacrimal_R D1%
10%
5%
0%
-5%
-10%
-15%
-20%
-25%
Percentage dose change
*
*
*
*
*
1
2
3
4
5
6
7
8
9
10
Patients

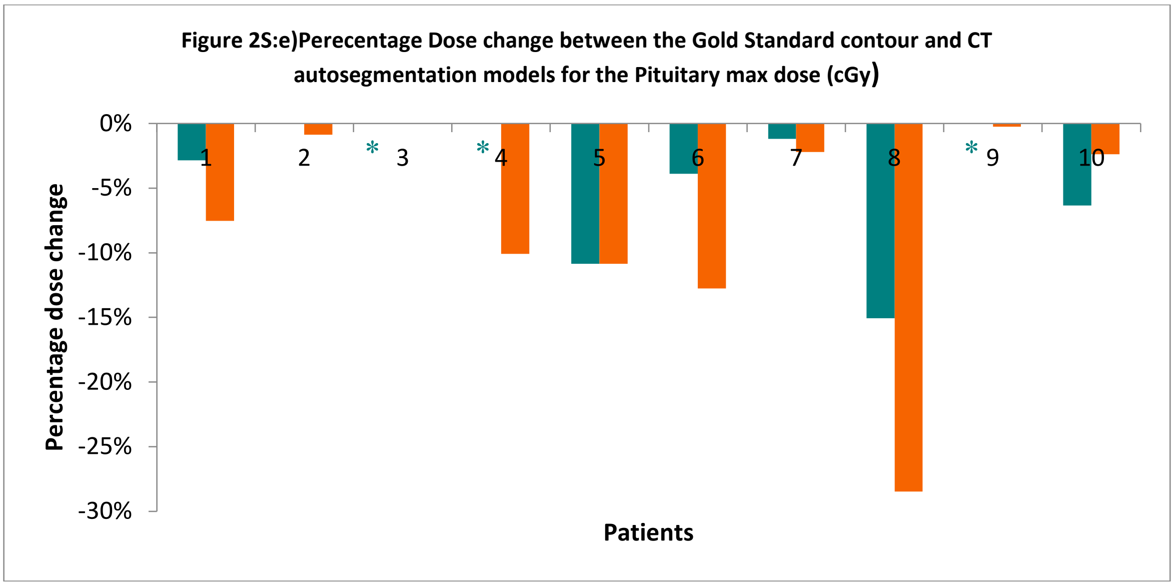
Figure 2S:e)Perecentage Dose change between the Gold Standard contour and CT autosegmentation models for the Pituitary max dose (cGy)
0%
-5%
-10%
-15%
-20%
-25%
-30%
Percentage dose change
1
2
* 3
* 4
5
6
7
8
* 9
10
Patients
