# Supplementary material for: Dosimetric impact of contour editing on CT and MRI deep‐learning autosegmentation for brain OARs
Source: J Appl Clin Med Phys. 2024 Apr 25;25(5):e14345. doi: 10.1002/acm2.14345 (PMC11087158; doi:10.1002/acm2.14345)
Supplement: Supplementary file 2 — Supplemental Information. [file ACM2-25-e14345-s003.docx]

***Supplementary Information***

- ***Tables:***

|  | | ΔBrainstem  D5% | ΔCochlea L  D50% | ΔCochlea  R  D50% | ΔGlnd  Lacrimal  L  D1% | ΔGlnd  Lacrimal  R  D1% | ΔLens  L  D1% | ΔLens  R  D1% | ΔOptic Chiasm  D1% | ΔOpticNrv  L  D1% | ΔOpticNrv  R  D1% | ΔOrbit  L  D1% | ΔOrbit  R  D1% | ΔPituitary  Max dose |
| --- | --- | --- | --- | --- | --- | --- | --- | --- | --- | --- | --- | --- | --- | --- |
| MRIeMRI*** vs MRIeCT | | | | | | | | | | | | | |  |
| P. Value  Threshold:  *p* ≤ 0.01 | | 0.318 | ** | ** | $$ | $$ | ** | 0.070 | 0.323 | 0.093 | 0.115 | 0.083 | 0.074 | 0.614 |
| Effect size:  Δ median | | 0% |  |  |  |  |  | -1% | 1% | 0% | 4% | 4% | 6% | 1% |
| N* | | 9 | 5 | 4 | 4 | 2 | 2 | 8 | 8 | 9 | 9 | 9 | 9 | 7 |
| MRIeMRI*** vs MRIu | | | | | | | | | | | | | | |
| P. Value  Threshold:  *p* ≤ 0.01 | | 0.965 | ** | ** | ## | ## | 0.435 | ** | 0.316 | 0.033 | 0.298 | 0.208 | **0.012** | 0.243 |
| Effect size:  Δ median | | 0% |  |  |  |  | 1% |  | 1% | 10% | 6% | 4% | 7% | 0% |
| N* | | 9 | 1 | 5 | 5 | 2 | 9 | 5 | 6 | 9 | 9 | 9 | 9 | 6 |
| MRIeCT*** vs MRIu | | | | | | | | | | | | | | |
| P. Value  Threshold:  *p* ≤ 0.01 | | 0.139 | $$ | ## | 0.318 | 0.263 | ## | 0.371 | $$ | 0.146 | 0.818 | 0.178 | 0.687 | $$ |
| Effect size:  Δ median | | 0% |  |  | 0% | -3% |  | 2% |  | 10% | 2% | 0% | 2% | 1% |
| N* | | 9 | 1 | 2 | 7 | 8 | 2 | 6 | 5 | 9 | 9 | 9 | 9 | 4 |
| * Number of compared segmentations (successfully segmented by both models considered)  ** MRIeMRI segmented more cases  $$ MRIeCT segmented more cases  ## MRI Unedited segmented more cases | | | | | | | | | | | | | | |

Table S1: Paired T-test results comparing dosimetric difference between the MRI models. Bold values indicate statistically significant differences (p ≤ 0.016). Insufficient successful segmentations were achieved by one of the models, this is noted ($$, **, or ##), indicating the superior model. A positive effect size indicates the tested model (***) was closer to the gold standard than the comparison model.

| CTeCT*** vs CTu | | | | | | | | | | | | | |
| --- | --- | --- | --- | --- | --- | --- | --- | --- | --- | --- | --- | --- | --- |
|  | **ΔBrainstem**  **D5%** | **ΔCochlea L**  **D50%** | **ΔCochlea**  **R**  **D50%** | **ΔGlnd Lacrimal**  **L**  **D1%** | **ΔGlnd**  **Lacrimal**  **R**  **D1%** | **ΔLens**  **L**  **D1%** | **ΔLens R**  **D1%** | **ΔOptic Chiasm**  **D1%** | **ΔOpticNrv**  **L**  **D1%** | **ΔOpticNrv**  **R**  **D1%** | **ΔOrbit**  **L**  **D1%** | **ΔOrbit**  **R**  **D1%** | **ΔPituitary**  **Max dose** |
| P. Value  Threshold: ≤0.05 | 0.139 | 0.250 | 0.872 | ** | ** | 0.692 | 0.359 | 0.097 | 0.695 | 0.269 | 0.461 | 0.147 | 0.163 |
| Effect size: Δ median | 3% | -2% | 1% | 1% | 1% | 0% | 0% | -7% | -8% | 0% | 0% | 3% | -4% |
| N* | 10 | 7 | 7 | 2 | 5 | 6 | 8 | 7 | 7 | 7 | 10 | 10 | 7 |
| * Number of compared segmentations (successfully segmented by both models considered)  ** CTeCT segmented more cases | | | | | | | | | | | | | |

Table S2: Paired T-test results comparing dosimetric difference between the CT models. Insufficient successful segmentations were achieved by one of the models, this is noted ($$, **, or ##), indicating the superior model. A positive effect size indicates the tested model (***) was closer to the gold standard than the comparison model.
