# Supplementary material for: Dosimetric impact of contour editing on CT and MRI deep‐learning autosegmentation for brain OARs
Source: J Appl Clin Med Phys. 2024 Apr 25;25(5):e14345. doi: 10.1002/acm2.14345 (PMC11087158; doi:10.1002/acm2.14345)
Supplement: Supplementary file 3 — Supplemental Information. [file ACM2-25-e14345-s002.docx]

***Supplementary Information***

- ***Training parameter information***:

The network consisted of a two-stage 3D-U-net design, where the first (localisation network) was used to extract smaller regions of the 3D image data for the second level models to refine the per-OAR segmentation.  The initial network used 4 maxpooling convolutional layers, with reLU activation, a 3*3 convolutional kernel and 24 filters at the initial layer. Dropout (p=0.5) and instance normalisation was applied to each layer.  Transposed convolution was used for up sampling.

Optimisation was performed using the Adam optimiser with learning rate 1*10-4 and a batch size of 1. Losses were computed using categorical cross entropy with Softmax activation.  Losses in the localisation model were weighted by inverse average OAR volume, to encourage localisation of small OARs. Data augmentation including translation, rotation and elastic deformation was applied during training.

- ***Dosimetric Evaluation: Statistical analysis:***

To evaluate the statistical significance of these metrics and determine the impact of editing before training the model, each test metric pair of the edited and unedited models was compared in each modality using the paired two-tailed Student’s t-test.

For the same patient, if the autosegmentation model failed to segment any OARs, and the comparable model was able to segment the missing OAR, this OAR was excluded from the pairwise comparison. A Bonferroni correction was applied to factor in a multiple-comparison correction used when several dependent or independent statistical tests are being performed simultaneously. (1 metric and 3 segmentation pairs for MRI, 1 metrics and one segmentation pair for CT). The Bonferroni corrected statistical significance threshold was *p* ≤ 0.01 (0.05/3) and ≤ 0.05 for the MRI and CT dosimetric evaluations, respectively.
